# Computational Pre-surgical Planning of Arterial Patch Reconstruction: Parametric Limits and *In Vitro* Validation

**DOI:** 10.1007/s10439-018-2043-5

**Published:** 2018-05-14

**Authors:** S. Samaneh Lashkarinia, Senol Piskin, Tijen A. Bozkaya, Ece Salihoglu, Can Yerebakan, Kerem Pekkan

**Affiliations:** 1 Department of Mechanical Engineering, Koc University, Rumeli Feneri Kampüsü, Sarıyer, Istanbul, Turkey; 2 Department of Mechanical Engineering, University of Texas at San Antonio, San Antonio, TX USA; 3 Department of Cardiovascular Surgery, Koc University Medical School, Istanbul, Turkey; 4 Department of Cardiovascular Surgery, Istanbul Medipol University, Istanbul, Turkey; 5 Cardiovascular Surgery, Children’s National Heart Institute, The George Washington University School of Medicine, Washington, DC USA

**Keywords:** Pulmonary outflow tract, PTFE patch, Pre-surgical planning, Congenital heart defects, Hemodynamics, Surgical materials, Rapid-prototyping, Computational biomechanics

## Abstract

Surgical treatment of congenital heart disease (CHD) involves complex vascular reconstructions utilizing artificial and native surgical materials. A successful surgical reconstruction achieves an optimal hemodynamic profile through the graft in spite of the complex post-operative vessel growth pattern and the altered pressure loading. This paper proposes a new *in silico* patient-specific pre-surgical planning framework for patch reconstruction and investigates its computational feasibility. The proposed protocol is applied to the patch repair of main pulmonary artery (MPA) stenosis in the Tetralogy of Fallot CHD template. The effects of stenosis grade, the three-dimensional (3D) shape of the surgical incision and material properties of the artificial patch are investigated. The release of residual stresses due to the surgical incision and the extra opening of the incision gap for patch implantation are simulated through a quasi-static finite-element vascular model with shell elements. Implantation of different unloaded patch shapes is simulated. The patched PA configuration is pressurized to the physiological post-operative blood pressure levels of 25 and 45 mmHg and the consequent post-operative stress distributions and patched artery shapes are computed. Stress–strain data obtained in-house, through the biaxial tensile tests for the mechanical properties of common surgical patch materials, Dacron, Polytetrafluoroethylene, human pericardium and porcine xenopericardium, are employed to represent the mechanical behavior of the patch material. Finite-element model is experimentally validated through the actual patch surgery reconstructions performed on the 3D printed anatomical stenosis replicas. The post-operative recovery of the initially narrowed lumen area and post-op tortuosity are quantified for all modeled cases. A computational fluid dynamics solver is used to evaluate post-operative pressure drop through the patch-reconstructed outflow tract. According to our findings, the shorter incisions made at the throat result in relatively low local peak stress values compared to other patch design alternatives. Longer cut and double patch cases are the most effective in repairing the initial stenosis level. After the patch insertion, the pressure drop in the artery due to blood flow decreases from 9.8 to 1.35 mmHg in the conventional surgical configuration. These results are in line with the clinical experience where a pressure gradient at or above 50 mmHg through the MPA can be an indication to intervene. The main strength of the proposed pre-surgical planning framework is its capability to predict the intra-operative and post-operative 3D vascular shape changes due to intramural pressure, cut length and configuration, for both artificial and native patch materials.

**Electronic supplementary material:**

The online version of this article (10.1007/s10439-018-2043-5) contains supplementary material, which is available to authorized users.

## Introduction

Most children who are born with a clinically significant congenital heart defect (CHD) require palliative congenital heart surgeries utilizing native and artificial patch materials for the reconstruction of the heart and great vessels. A typical example of patch reconstruction surgery is the repair of vascular anomalies of the right side, as in the Tetralogy of Fallot (TOF) disease.^2^,^13^ In these operations, through the relief of main pulmonary artery (MPA) stenosis with an arterial patch, a balanced PA flow distribution is desired, which is influenced by the post-surgery conduit geometry and pressure levels. This task is further complicated by the large variability in patient-specific anatomy and the PA size. Unfavorable post-operative pulmonary hemodynamics may further result in abnormal pulmonary vascular remodeling.^21^ Therefore, the main objective of the present study is to develop a pre-surgical patch planning and biomechanical performance prediction system for TOF surgeries. It is hypothesized that this tool will assist the surgical team to achieve the best patch-reconstructed MPA conduit flow-pattern and mechanical stress customized for the individual patient *in silico*.

While the framework demonstrated in this paper is for MPA reconstruction, the methodology is equally applicable to the aortic patch repair surgeries (such as aortic coarctation and hypoplastic arch) with modifications on vessel dimensions, material property models and pre- post- operative loading.

Pre-surgical hemodynamic planning^15^,^43^ integrated with the 3D rapid-prototyping technology^12^ has emerged as a useful tool in the surgical management of the complex congenital cardiovascular defects.^29^ Our recent investigations on the hemodynamic performance of right ventricular outflow conduits, suggested an improved performance when customized valve leakage area and orientation are considered.^11^ Following the success in patient-specific computational fluid dynamics (CFD) simulations,^1^,^27^,^35^ the pre-surgical planning concept, based on soft-tissue finite element models (FEM) has also been demonstrated.^3^,^36^,^37^,^41^,^42^ Particularly, the implementation of FEM in the pre-surgical planning of complex heart valve repair procedures is well-established.^4^,^23^,^30^^–^32,44 Likewise, computational soft-tissue models are essential in the evaluation of stenting procedures of aorta, pulmonary and carotid arteries.^8^,^25^,^33^ A relevant study by Tang *et al.*^36^ have investigated the effects of flow and stress–strain distributions in dilated right ventricle (RV) of a TOF patient using FEM method. Their findings showed that, artificial patches with similar material properties as the native RV tissue and small size lead to better RV function and recovery. This knowledge lead to an alternative RV patch strategy for contracting myocardium having decreased stress levels on the patch, improved RV function and reduced patch area.^36^,^42^

In this paper, we present a pre-surgical, computer-aided surgical patch design framework for great arteries having arbitrary 3D stenosis sections. The proposed framework is validated through the actual surgical patch reconstructions performed on rapid-prototype replicas (Supplementary Material 1). This approach allows us to predict the intra- and post- operative anatomy, mechanical loading and the hemodynamics of the surgical reconstructions. It also allows the structural optimization of the reconstructed patch region before the surgical execution, leading improved performance. Particularly, pre-surgical planning of the 3D patch shape will reduce cardiopulmonary bypass time and consequently could influence the probability of post-operative complications.^16^,^38^

## Methods

The effect of geometric parameters in the proposed pre-surgical patch design framework is studied through a realistic main pulmonary artery segment having a symmetric stenosis that needs repair. A symmetric MPA stenosis is clinically the most common case. The model’s anatomical dimensions correspond to a 9 year-old child’s MPA that was taken from reference values reported in a statistical clinical study.^19^ Model has an inner diameter of 18 mm and 1 mm uniform vessel wall thickness. Two stenosis levels of 70 and 80% in vessel cross-sectional area, which are typically repaired through a patch implantation, are considered as an indication for surgical intervention. The dimensions of the initial stenosed, pre-operative loading state is in agreement with our earlier study that employed clinical patient MRI and CT scans,^11^ typically operating at a mean intramural blood pressure of 60 mmHg for Tetralogy of Fallot.^22^

**Figure 1:**
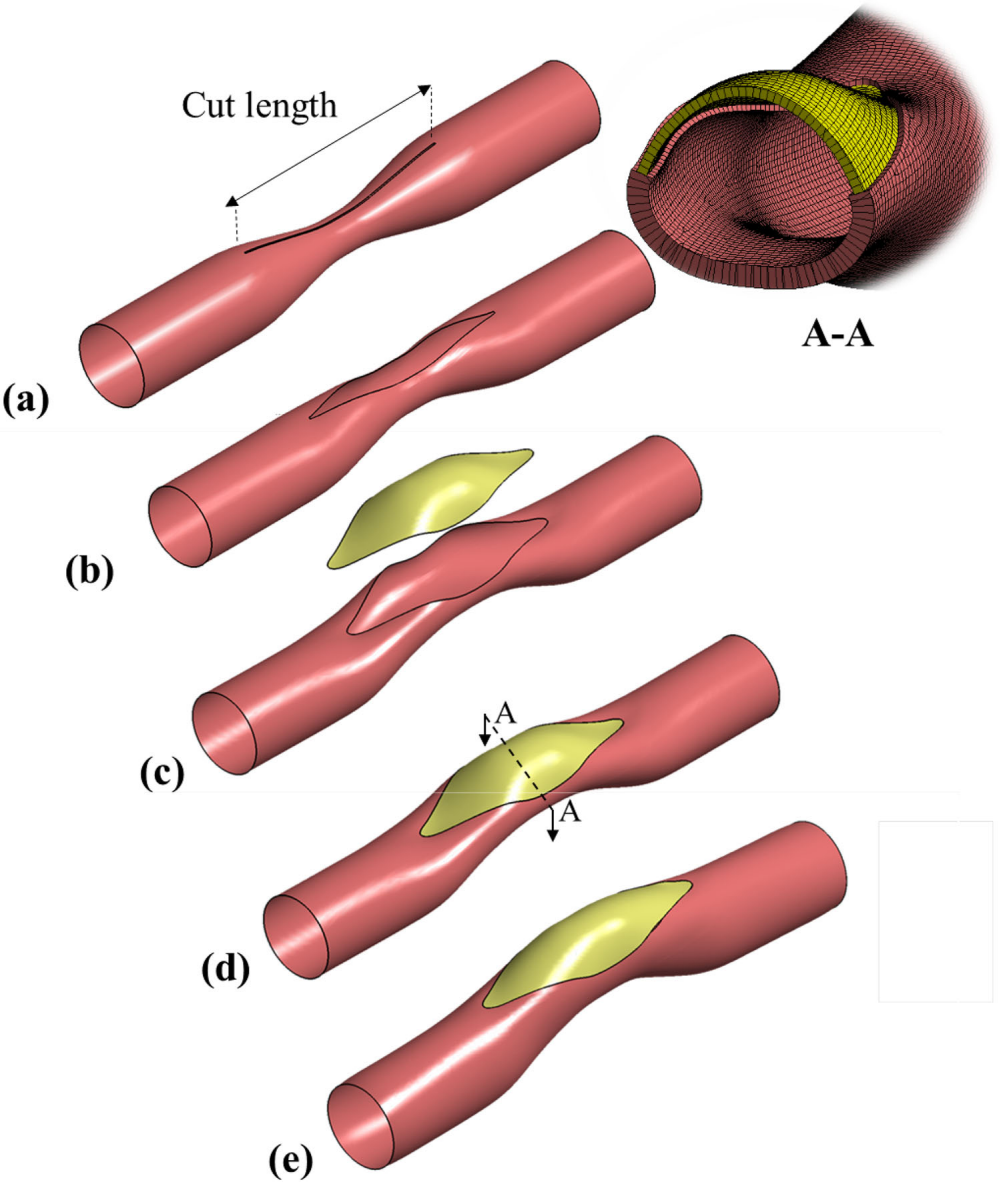
An idealized main pulmonary artery having symmetric stenosis leading to an asymmetric post-operative conduit after patch repair. The sequence of virtual surgical instances is illustrated in sequence. (a) Cutting an incision slit on the artery, (b) load-free state due to the release of residual stress after the incision, (c) intraoperative stretching of the incision gap for enlarging and deforming an initially flat patch to 3D shape that is tangent to the suture line curvature, (d) the suturing of patch to the artery (e) following cardiopulmonary bypass, the arterial pressure is established and the reconstructed PA conforms to its acute post-operative shape.

The pre-surgical patch design framework consists of multiple simulation steps partially summarized in Fig. 1. The sequence of surgical operations or processes simulated through the finite element model, starts with the reduction of intramural pressure to the zero level, due to the cardiopulmonary by-pass and aortic cross-clamp. At this stage, only the residual stresses remain in the artery, which could be estimated prior to the operation non-invasively, if needed.^10^ The residual stresses are introduced through the “pressure-equivalent residual stress” technique where a finite intramural pressure corresponding to the desired residual stress distribution is applied to the unloaded configuration,^10^ which is typically 5 mmHg. Along the narrowed arterial segment, the surgical incision is introduced as a surface curve created interactively through an anatomical editing tool (Geomagic Inc, NC, USA)—see Fig. 1(a). This 3D curve of incision is converted to a zero-gap slit in the FEM model where the cross-sectional area of the cut in both sides are opened slightly due to the release of residual stresses—see Fig. 1(b). Next, an extra pull, if required by the surgeon is represented through a distributed traction boundary condition, normal to cut surface of the vessel—see Fig. 1(c). The width of the opened region depends on the magnitude and direction of the force applied by the surgeon and the residual stress level. This gap, which is prepared just before the patch-implantation stage, can be precisely controlled in the current surgical procedure, as this opening is an important intra-operative geometrical parameter. To cover this gap, an initial patch shape needs to be selected and there are several alternatives explored in the present work (please see Discussion). However, in order to standardize this process for the different parametric cases considered, a “tangent patch” that matches the local curvature of surrounding native tissue is employed (Geomagic Inc, NC, USA)—see Fig. 1(c). This patch is then sutured to the native artery along the mid-artery layer by creating a bonded connection in the FEM model (ANSYS Inc, Somerset, PA, USA)—see Fig. 1(d).

In order to simulate the post-operative stage after the heart is re-introduced to the circulation (following cardio-pulmonary bypass) and to simulate the performance of the designed patch, the patched PA configuration is pressurized to the typical intramural post-surgery blood pressure levels of 25 and 45 mmHg—see Fig. 1(e),^22^ typically measured *via* routine cardiac catheterization.

Ten different scenarios have been simulated in the present study as detailed in Table 1. Case *Baseline* represents the conventional surgical configuration as implemented in clinics, where a straight slit, equal to the stenosis length is made on the artery. This is chosen as the nominal case and the simulation results of the other cases are compared to the *Baseline* case. To investigate the effects of various geometric parameters, the arteries with 70% stenosis level are cut by three different incision lengths; equal to (*Baseline*), shorter (*Length_1*) or longer (*Length_2*) than the stenosis length. Different slit shapes, such as oblique (*Shape_1*) and straight cut (all other cases) on the artery are also tested to determine the optimized shape of the patch. *Shape_2* represents the double cut and double patched scenario. Case name *Stenosis* represents 80% stenosis level with a straight cut equal to the stenosed region’s length. The effects of patch material and thickness on reconstructed PA’s post-operative performance are investigated through *Material* group cases. Table 2 details the properties of all materials used in the present study. Finally, case *Pressure* presents the patched artery with different post-operative blood pressure.

**Table 1:** Simulated patch scenarios and their corresponding geometric parameters.

Group name	Case number	Cut length (mm)	Cut shape	Slit stretch (mm)	Patch material	Stenosis percentage	Applied post-operative pressure (mmHg)
Baseline	–	50	Straight	16	*PTFE*	70	25
Length	1	40	Straight	16	*PTFE*	70	25
	2	60	Straight	16	*PTFE*	70	25
Shape	1	50	Oblique	16	*PTFE*	70	25
	2	50	Double-Straight	16–16	*PTFE*	70	25
Stenosis	–	50	Straight	16	*PTFE*	80	25
Material	1	50	Straight	16	*Human pericardium*	70	25
	2	50	Straight	16	*Porcine xenopericardium*	70	25
	3	50	Straight	16	*Dacron*	70	25
Pressure	–	50	Straight	16	*PTFE*	70	45

**Table 2:** Linear elastic material properties and patch thickness of different surgical materials commonly used in pediatric cardiovascular operations obtained from in-house biaxial mechanical tests *except, the Main Pulmonary Artery Human—9 year old which is provided from Wagenseil *et al.*’s study.^39^

Material	Poisson’s ratio (–)	Young’s modulus (MPa)	Thickness (mm)
Glutaraldehyde treated porcine xenopericardium	0.39	2.89	0.4
Fresh human pericardium	0.4	3.4	0.5
PTFE	0.31	1.4	0.7
Dacron	0.42	1.19	0.6
Main pulmonary artery human—9 year-old*	0.45	0.75	1.0

A quasi-static finite element model is employed using high-quality tetrahedral shell elements (ANSYS Inc, Somerset, PA, USA). Mesh convergence runs are performed with edge lengths of 50–150 *µ*m and a relatively finer grid-size (100 *µ*m) is chosen. The sensitivity of results to different boundary condition schemes is tested to replicate the intra-operative clinical characteristics. For example, fixing both vessel ends at all degrees of freedom, lead to excessive patch deformations that have not been observed clinically. Thus, the simulations are performed by completely fixing the boundary close to the outflow tract, while at the other end, free-movement in the axial direction is specified. The selected boundary condition scheme closely described the surgical state based on the clinicians input.

The finite element solver is validated experimentally, where the entire sequence of pre-operative surgical actions are replicated using a flexible rapid-prototype stenosis model (please see Supplementary Material 1). The rapid-prototype replica of *Baseline* case is patched using a Polytetrafluoroethylene (PTFE) material (Hemashield Gold Knitted Double Velour Vascular Graft, Maquet Getinge group, Rastatt, Germany) and tested in a mock-up static pressure set-up. The error in computed deformations is less than 2.7%, compared to the experimental post-op measurements.

CFD analysis is performed using commercial software ANSYS Fluent 17.0 (ANSYS, Inc, Somerset, PA, USA). Simulations are first run with a grid size with the 500 *µ*m edge length. Based on a grid convergence runs targeting a constant pressure drop throughout the patched vessel, a grid size of 200 *µ*m, leading to 10^−3^ Pa pressure drop difference, is chosen. The CFD software is configured to implement an Algebraic Multi-grid (AMG) scheme to accelerate the convergence of the incompressible Newtonian solver and employ a second-order accurate discretization scheme.^10^,^20^,^26^,^40^ The cardiovascular solver has been validated experimentally in our earlier studies.^5^ Velocity inlet boundary conditions are employed having a plug flow velocity profile (which is typical for outflow tracts) at 4 LPM mean pulmonary flow rate.^6^ Zero pressure outlet boundary condition is assigned at the vessel exit. The calculated average Reynolds number varies in 1531–1758 range along the vessel for post-surgery cases. For the pre-surgery case (70% stenosis), the average Reynolds number reaches 2607. Therefore, a direct numerical simulation (DNS)-like transient laminar flow model is implemented in the flow simulation of all cases. All flow simulations are continued until convergence of 10^−5^ residue. We reported converged running average results for pressure. To reach fully developed flow at the inlet and overcome the downstream end-effect on the model, the inlet and outlet are extruded accordingly while just the main geometry is shown in Figures.

The state-of-art cardiovascular patch materials conventionally used in pediatric surgeries include Dacron (Polytetrafluoroethylene Gore-tex Stretch Vascular Graft, Gore, Arizona, USA), PTFE (Hemashield Gold Knitted Double Velour Vascular Graft, Maquet Getinge group, Rastatt, Germany), Glutaraldehyde treated porcine xenopericardium and fresh or glutaraldehyde treated human pericardium. Biaxial mechanical tests are conducted for each material and corresponding stress–strain data are obtained by sinusoidal stretching of square shaped samples (10 × 10 mm) up to 20% in both axial directions, using four linear motor configurations in the BOSE planar biaxial test system (BOSE, Framingham, Massachusetts). Strains are measured in both directions through the tissue dye-marked fiducial points as well as linear motor positions. The linear elastic material properties (Poisson’s ratio and Young’s modulus) are extracted from this data through a multi-dimensional least square fit corresponding to the operating range of the MPA^9^—Please see Supplementary Material 2 for details.

Finite element analysis shows that the thickness of the patch is an important parameter that affects the post-operative performance and shape of the patched artery critically. To overcome conventional measurement errors, the wall thickness of the Glutaraldehyde treated porcine xenopericardium, fresh human pericardium and artificial patches are measured using an optical coherence tomography (OCT) system (Thorlabs Inc, NJ, USA). Table 2 shows the extracted material properties and thickness of the tested graft tissues. As such, the material properties of the main pulmonary artery are assumed to be almost incompressible linear elastic.^39^ Young’s modulus and Poisson’s ratio values are reported in Table 2.

Finite element and CFD simulations enabled the computation of multiple biomechanical performance indices, which are employed here to evaluate and compare the different patch designs and intra-operative strategies. These performance indices are listed below:

- Intra-operative stress introduced on the vessel due to opening of slit.
- Post-operative, equivalent von Mises stress distribution and gradients of the native artery/artificial patch providing maximum post-operative stress levels.
- Post-operative total deformation of patch and native artery.
- Post-operative stress gradients at the suture line due to material mismatch.
- Percentage of post-operative recovery of the initial stenosed lumen area and the corresponding hemodynamic post-operative pressure drop through the repaired anatomy.
- Post-operative arterial tortuosity; offset deviation of vessel centerline from the initial MPA pathway.

For the last two performance parameters, the cross-sectional areas normal to the centerline of artery for pre-surgery and post-surgery conditions are obtained using the vascular modelling toolkit library.^1^ Achieving larger cross-sectional areas through the reconstructed surgical pathway is essential for optimal blood flow distribution in the PA. Local percentages of stenosis along the centerline are calculated by dividing the cross sectional area at the specific point to the maximum cross sectional area in model in post-operative situation. Tortuosity of the arterial model in post-operative condition is also measured in terms of the centerline’s offset from the initial MPA’s centerline at the throat.

## Results

**Figure 2:**
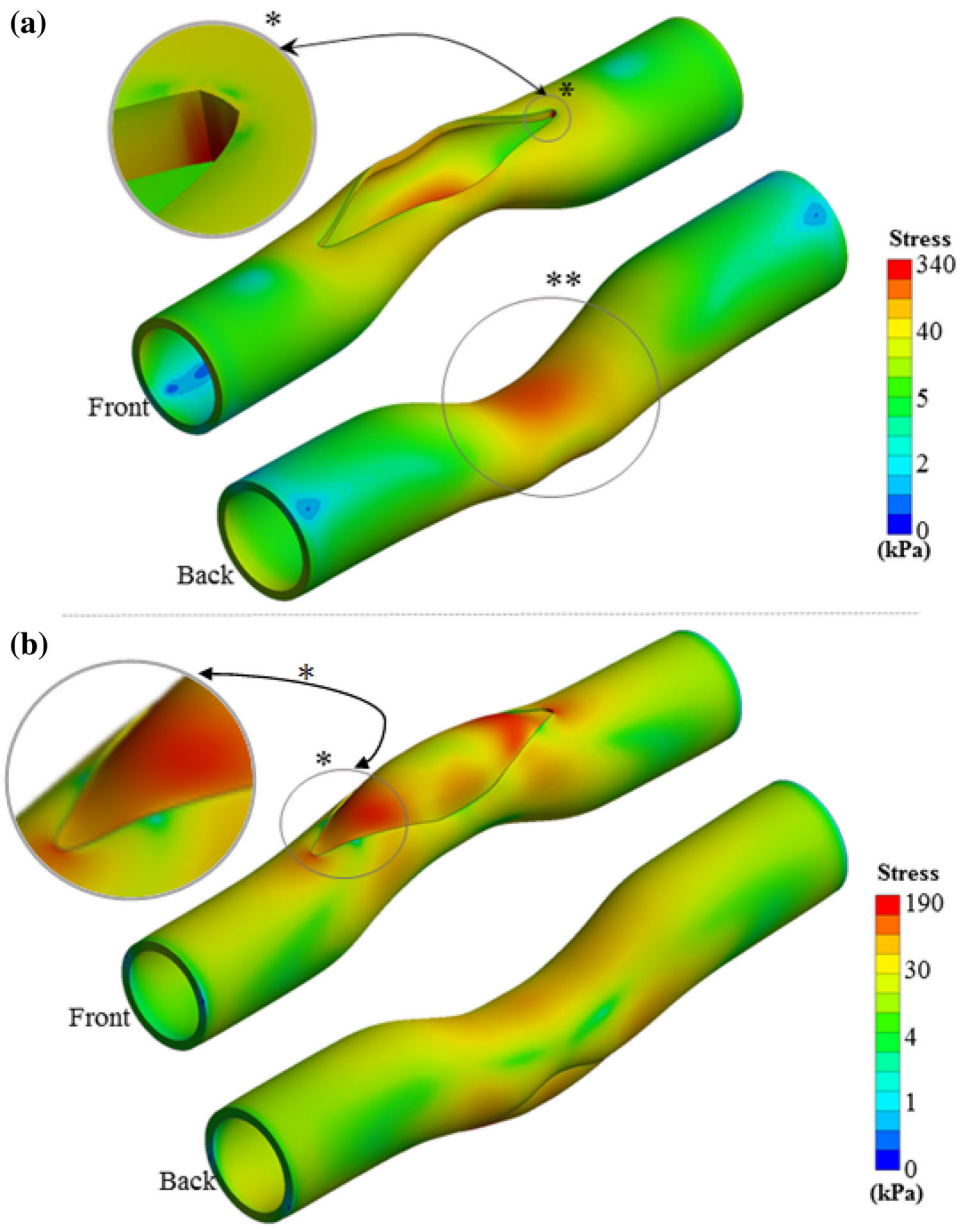
(a) Equivalent stress distribution on the artery during the opening of slit in surgery. * indicates the highest stressed region of the main pulmonary artery, which also observed symmetrically at the opposite side of the vessel. ** Highlights the relatively moderate stressed regions. (b) Equivalent stress distribution on the patch and artery after pressurizing to post-operative blood pressure level. *Shows the stress concentration regions on the patch and the artery. Stress gradient maps are shown in logarithmic scale to highlight the vessel and patch connection region’s stress contours in various colors.

The equivalent stress distribution on the native MPA for the case *Baseline* is plotted in Fig. 2(a), immediately after that surgeon has opened the slit to enlarge the patch implantation gap. Stress concentrations at this intra-operative stage occur at both ends of cut native tissue. Another region with high stress levels (300–330 kPa) is observed at the initial stenosis throat. The relation between the width of the opening and the stress level is nonlinear. For example, when the model with 10% higher stenosis level (case *Stenosis*) is compared to case *Baseline*, the average stress around the gap increases 40% for obtaining a 16 mm-wide initial gap opening in both cases.

Figure 2(b) shows the stress distribution on the native artery for case *Baseline* after the patch implantation and pressurization (corresponding to the early post-operative stage). Maximum stress occurs at areas proximal to the stitch on the artery reaching 100–190 kPa levels. Patch tips also experience 63% of maximum stress on the artery while the un-patched native side of the stenosed region has moderate stress levels (30–80 kPa). All simulated cases predicted similar stress distribution on the artery and patch, but with considerable differences in stress levels.

**Figure 3:**
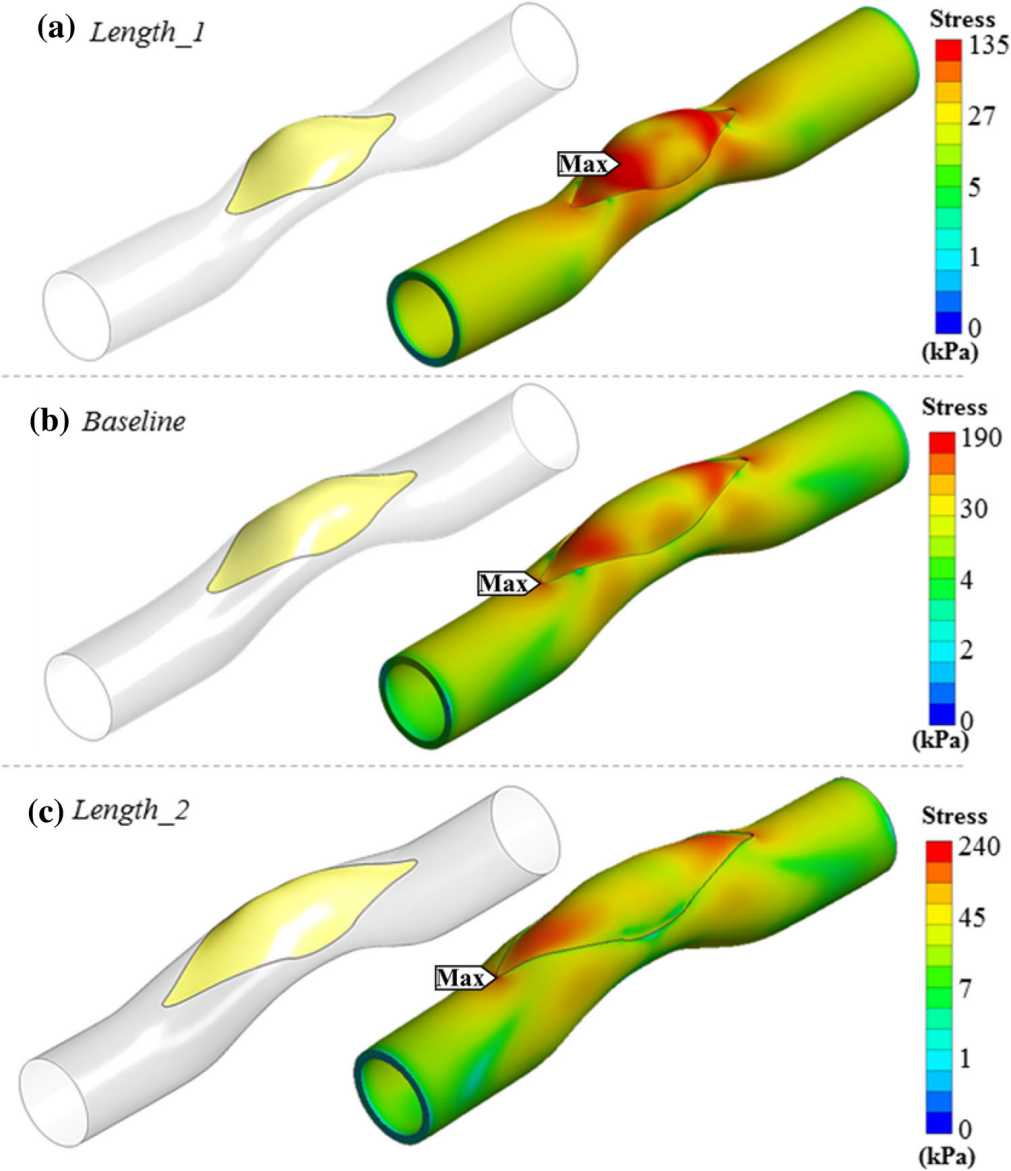
Comparison of von-Misses stress distributions on patched models having different surgical incision lengths. Left column shows the generated patch for three different cases - right column shows equivalent stress distribution on patch and artery, considering post-operative blood pressure. (a) 20% shorter cut than stenosis length (*Length_1*). (b) Stenosis length same as the cut length (*Baseline*). (c) 20% longer than stenosis length (*Length_2*). Stress gradient maps are shown in logarithmic scale to highlight the vessel and patch connection region’s stress contours in various colors.

Figure 3 shows the 3D post-operative shape of designed patches for the cases in *Length* group. The effect of slit length on the post-operative shape of the patch is examined using the results of these cases. All patches are designed for a standard 16 mm-width initial gap opening. For the shorter slit length, a patch area of 488 mm^2^ is required which increases to 610 and 807 mm^2^ for *Baseline* and *Length_2* cases, respectively. Likewise, the maximum stress value on these models increases from 135 to 239 kPa as the slit length is increased from 4 to 6 mm, respectively. For the case *Baseline* and case *Length_2*, maximum stress occurs at the artery while for case *Length_1,* the implanted patch bears the highest stress. As the slit length decreases, high stress regions extend further such that they overlap with the highly deformed cambered center region of the patch.

**Figure 4:**
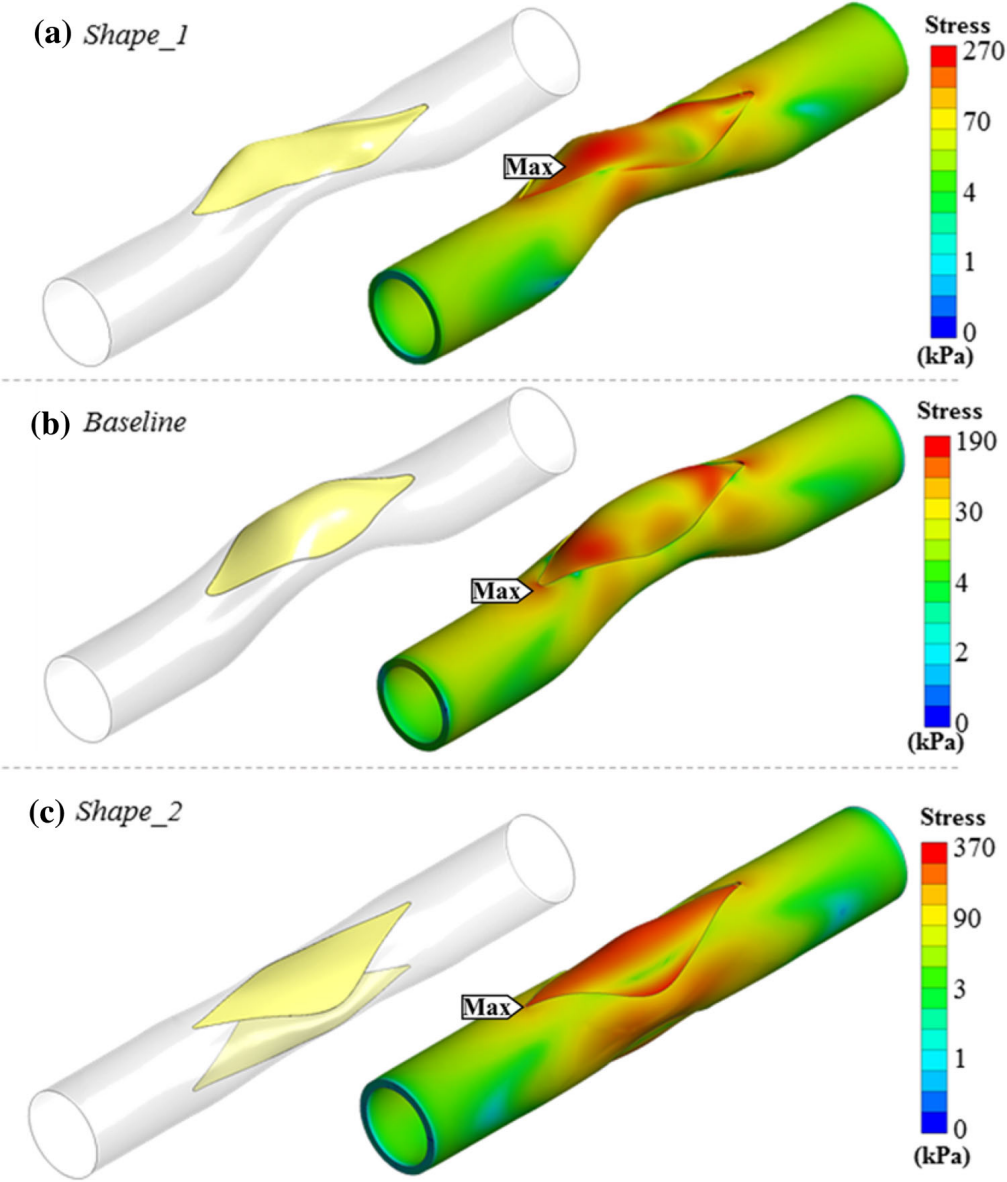
Exploring novel surgical options in main pulmonary artery patch surgery using the proposed simulation framework. (a) An oblique slit having a helix angle of 5 degree measured from center-line is introduced (*Shape_1*). (b) Straight cut on the artery (*Baseline*). (c) Implanting two similar patches on both sides of the artery (*Shape_2*). Stress gradient maps are shown in logarithmic scale to highlight the vessel and patch connection region’s stress contours in various colors.

Simulation results for two novel patch configurations, which are not used conventionally in clinics, are presented in this section—see Fig. 4. The first patch configuration is obtained through an oblique cut at the stenosis region (*Shape_1*). The 3D tangent patch generated for this case is more distorted in post-op state compared to the standard straight cut case *(Baseline)*. Stress level on the patch was found to be 80% higher for case *Shape_1* compared to the case *Baseline,* while the patch surface area decreased 16%.

Implementing the patch to the stenosis region by using a single vessel opening recovers the narrowing of the vessel partly and remains the second half stenosed as shown in Fig. 2(b). To overcome this residual vessel narrowing effect, one approach is to patch both sides of the vessels with two identical patches instead of one as shown in Fig. 4(c). Indeed, the resulting post-operative patch reconstructions are relatively flatter compared to the single patch solution and are able to recover the stenosis almost fully on both sides of the artery. However, the total patch surface area increases 67% and the maximum stress on the patch rises from 119 to 216 kPa. Native vessel section also bare 95% higher maximum stress value compared to the *Baseline* configuration.

**Figure 5:**
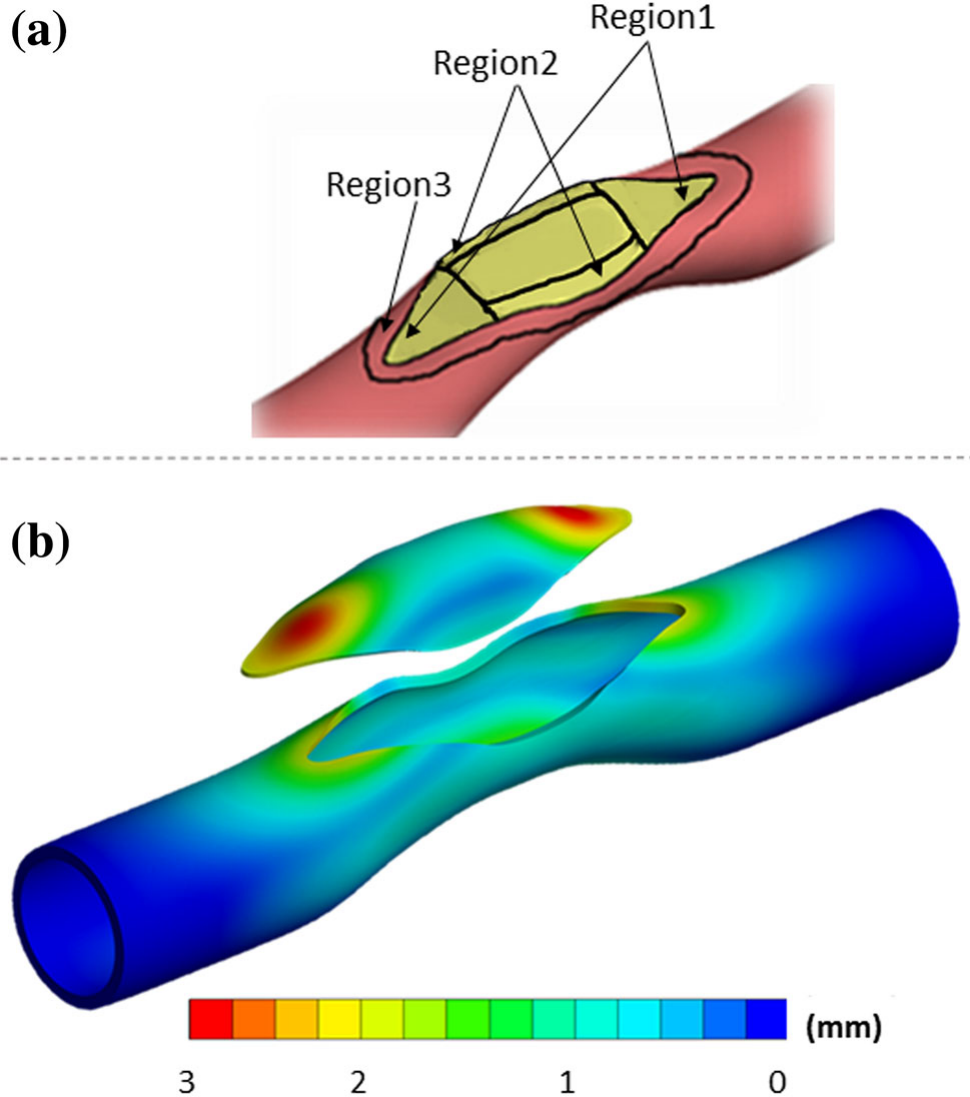
(a) Three major regions of patch and artery that experience different mechanical loading. (b) These regions are identified based on the corresponding stress patterns. For example, the total deformation on patched artery (*Baseline*) at the post-operative state are presented in the figure (patch and artery are shown separately).

Simulation results for patch configurations with different materials are presented in this section. The mechanical behaviors of four different patch materials are examined. Three critical zones in terms of highest and lowest total post-operative deformation are identified in order to present the results in a compact form, as shown in Fig. 5(a).

**Figure 6:**
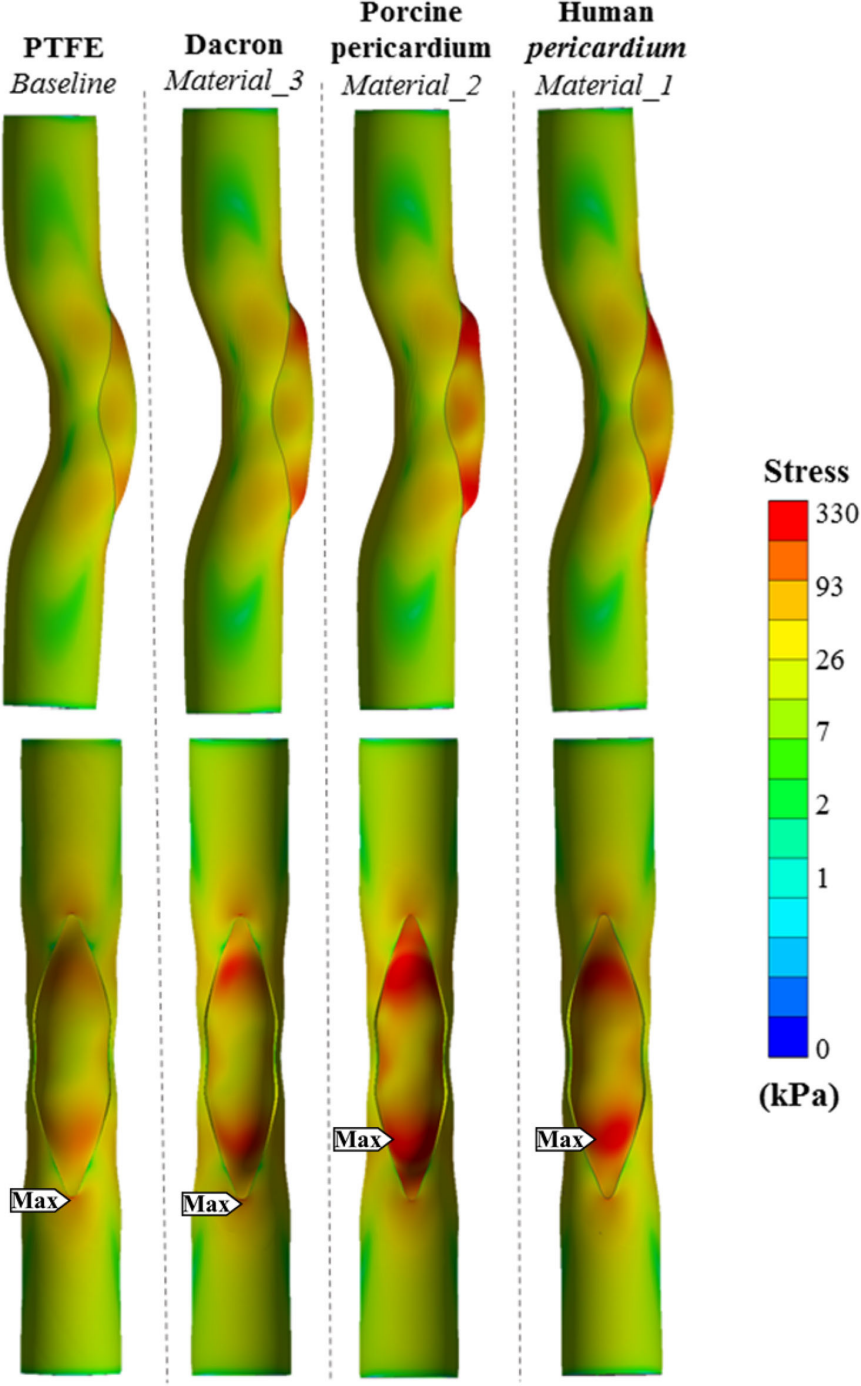
Equivalent stress distributions after patch reconstruction corresponding to the acute post-operative state, for different patch materials. Top row shows the coronal view of parametric material models and bottom row shows the corresponding sagittal view. Stress gradient maps are shown in logarithmic scale to highlight the vessel and patch connection region’s stress contours in various colors.

Figure 5(b) shows the computed deformations for the case *Baseline* as an exemplary of all material cases. Other patch materials lead to similar total deformation distributions and the highest deformation regions are localized proximal to the ends of the patch (Region 1 in Fig. 5(b)). Although the deformation distributions are similar, stenosis recovery ratios vary for different materials: 46% (PTFE), 45% (human pericardium), 43% (porcine xenopericardium) and 41% (Dacron). Figure 6 shows these deformation and stress distribution.

Since the wall thickness of available patch materials are measured to be significantly different (Table 2) additional model verification studies are conducted where the sensitivity of patch thickness on post-operative mechanical loading is established. For example peak total deformation on patch corners decreases from 5.1 to 3.6 mm when the thickness of patch is increased from 0.6 mm Dacron patch (Material_3) to 0.7 mm, while peak stress on the model decreases slightly from 198 to 191 kPa. Other interesting comparison case is the modeling of human pericardium tissue with 0.4 mm thickness instead of its measured thickness of 0.5 mm. In this case, the peak total deformation and maximum stress value on the model is increased from 3.1 to 4.8 mm and from 255 to 319 kPa respectively. These simulations indicate that besides the material properties, the thickness of the patch affects total deformation values significantly that can result in local “bumpy” regions with high deformations, proximal to patch corners as shown in Fig. 6. These local bumps are visible in cases Material_2 and Material_3, in which a maximum total deformation of 5.7 and 5.1 mm respectively, occurs in bumpy peaks close to the patch corners (Region 1 in Figure). For human pericardium (Material_1) and PTFE (Baseline), the post-op patch shape is entirely smooth at the ends.

Maximum equivalent stress occurs at the ends of the graft next to the stitched place (Regions 1 and 2) in all patch material cases. Both biological grafts (*Material_1* and *Material_2*) have larger maximum stress values on the patch (255 and 325 kPa, respectively) compared to artificial grafts (*Baseline* and *Material_3*). On the other hand, first and second maximum stress values of 190 and 198 kPa occur around the stitch area of the artery (region 3) in *Baseline* and *Material_3* (artificial grafts cases), respectively.

The lowest stress difference (maximum equivalent stress) between the patch and the artery occur in the PTFE patch (*Baseline*), along the stitch lines, reaching 21 kPa. Whereas the corresponding stress level is significantly higher (171 kPa) in the porcine xenopericardium patched case (*Material_2)*.

**Figure 7:**
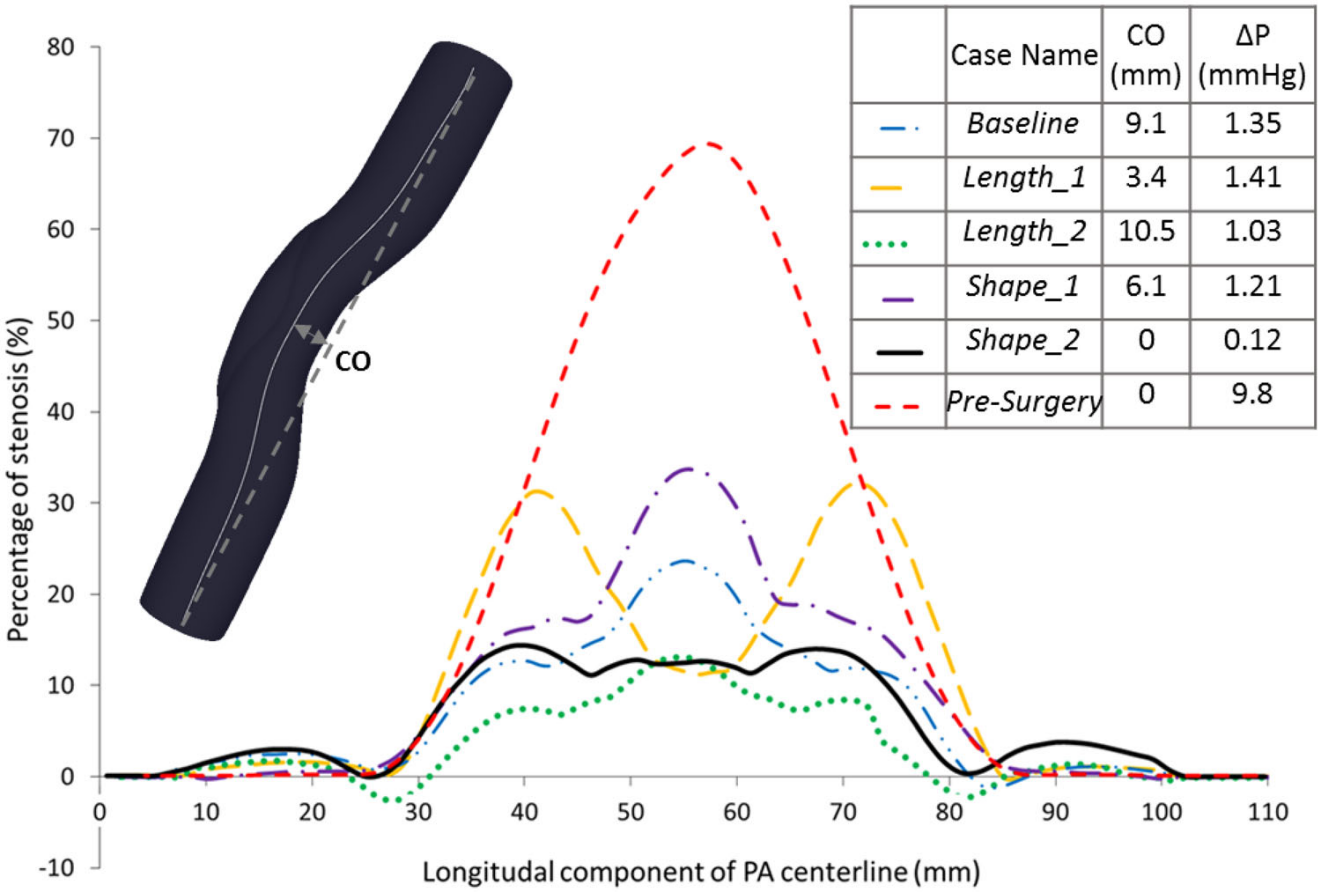
The local percentage of stenosis along the centerline is compared between pre-surgery case (70% stenosis level) and the post-operative cases. Pressure drop values and centerline offset (CO) at the throat are labeled near the case names.

The post-operative cross-sectional area variation along the axial length is plotted for groups *Baseline, Length* and *Shape* in Fig. 7. These cases employ to the same baseline surgical patch material (PTFE). Please note that the lumen area recovery characteristics for different patch materials are discussed earlier.

All cases in groups *Baseline*, *Length* and *Shape* display an increasing–decreasing trend where the throat corresponds to the maximum post-operative residual stenosis location, except the shorter cut model (*Length_1*). Interestingly, the *Length_1* case displays a more complex post-operative vessel area variation. In this case, the pre-operative throat location is the most enlarged section post-operatively (9% stenosis is remaining) while the minimum lumen cross-section is observed at the patch ends where 32% stenosis is remaining. These narrow regions, make the case *Length_1* disadvantageous in lumen area recovery point of view. In Fig. 7, the green dotted line represents the longer cut (*Length_2*), which lies under all other lines. So, the least local residual stenosis remains for the case *Length_2.* A negative percentage of stenosis recovery (down to − 4%), which means dilation of the artery at the patch end, is observed only for this case.

Post-operative cross-sectional area variations are computed for the other cases, but not shown here for the sake of brevity. In both artificial and natural material grafts, there is a slight difference in cross section areas.

The effect of pressure change on stenosis recovery is also tested in the current study. It is observed that an 80% increase in post-operative pressure (*Pressure* compared to *Baseline*) causes 29% decrease in the percentage of final post-operative stenosis as shown in Table 3.

**Table 3:** The performance parameters for the 10 simulated cases are summarized.

Case name	Patch area (mm^2^)	Post-operative stenosis (%)	Max arterial stress (kPa)	Max patch stress (kPa)	Average patch stress (kPa)
Baseline	610	24	189	119	40
Length_1	488	32	81	135	45
Length_2	807	13	239	140	34
Shape_1	514	34	52	268	72
Shape_2	511–511	14	369	216	104
Stenosis	535	39	176	127	42
Material_1	610	25	151	255	39
Material_2	610	27	154	325	39
Material_3	610	29	198	177	40
Pressure	610	17	457	250	81

**Figure 8:**
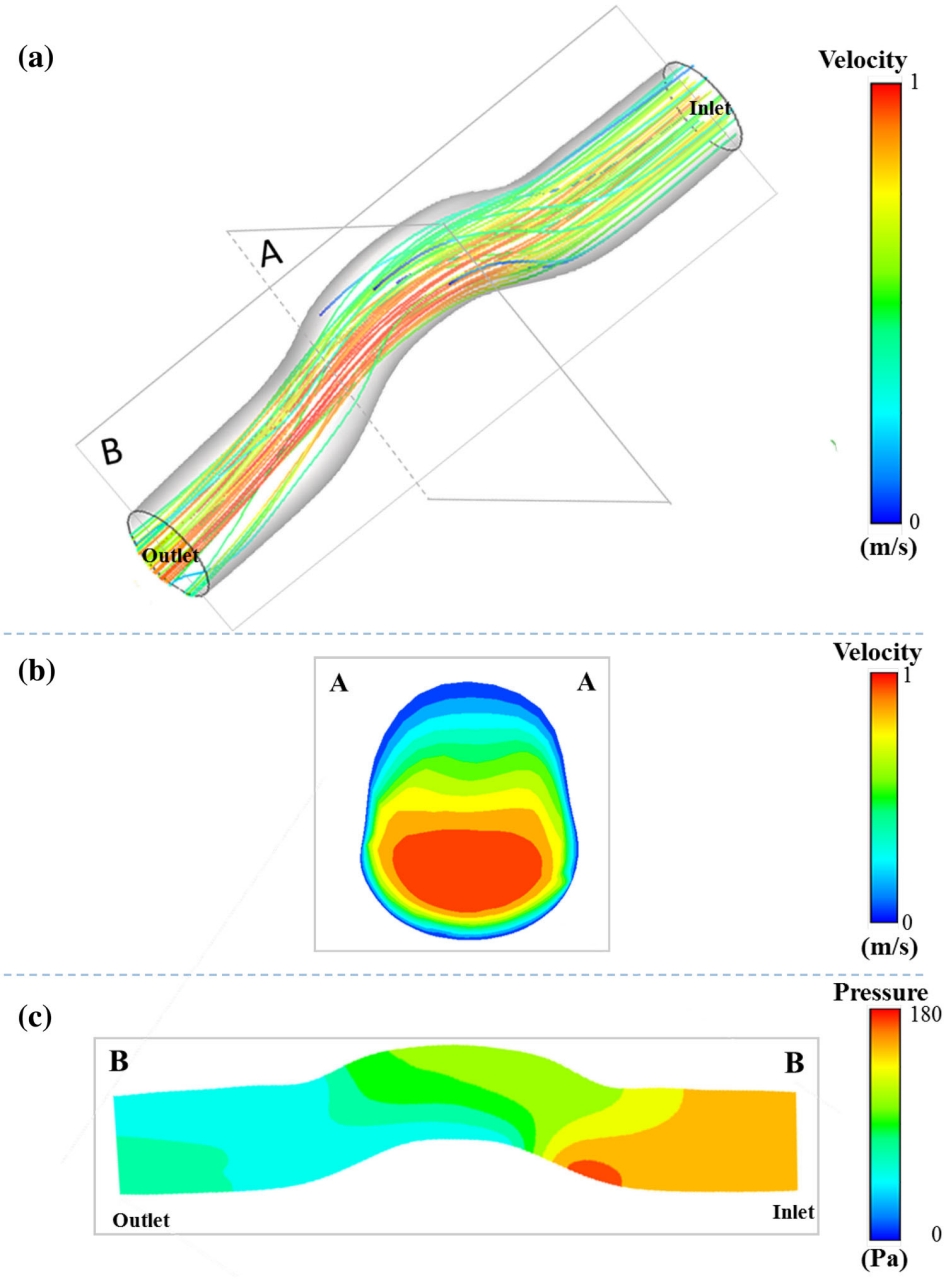
Patch hemodynamics quantified through the computational fluid dynamics analysis to compute pressure drop values. Only the *Baseline* case results are provided for brevity or s of other cases please refer to Fig. 7. (a) Velocity streamlines released from the inlet are colored by blood velocity magnitude. (b) Velocity magnitude contours of through the cross sectional cut of the vessel throat region (labeled by A–A). (c) Pressure distribution contour throughout the longitudinal direction at the mid-plane of the model (labeled by B–B).

Figure 8 presents the visualization of the blood flow through the patched pulmonary artery lumen in the *Baseline* case in terms of pressure, streamlines and velocity magnitude. A pressure difference of 1.35 mmHg from the inlet to the outlet of the vessel is computed. Due to the tortuosity of the patched conduit, the radial pressure variation is observed at the patched region. Cross sectional velocity distribution at the patched throat region of the vessel demonstrated a shift in the peak flow (~ 1 m/s magnitude). This shift persists downstream of the patch region.

Pressure drop and post-operative centerline offset values for three different slit length cases are shown in Fig. 7. The most torturous case is the longer cut case (*Length_2*), which has 6.1 mm more centerline offset at the throat compared to the shorter cut case (*Length_1*). As expected, there are drastic changes in pressure-drop from 9.8 mmHg in pre-surgery case to 1.35 mmHg in *Baseline* post-operative case. In comparison between post-operative cases, pressure drop in the *Baseline* case decreases 0.32 mmHg when incision length is increased 10 mm in the case Length_2, while 10 mm decrease in cut length (*Length_1*) causes just an additional 0.06 mmHg pressure drop in the model.

## Discussion

Patch reconstruction is a fundamental technique employed in majority of surgical interventions of congenital heart diseases. For the surgeon, the prediction of the post-operative shape of the patch and the native tissue is a complicated task. The use of a patch design approach that provides customized, patient-specific geometries, as proposed in this paper will enable clinicians to rapidly implement a planned intervention on a 3D computer model systematically, visualize its resulting structural stresses, compute hemodynamic and mechanical performance indices and predict the 3D vascular immediate post-operative anatomical shape of their intended surgery without *in vivo* execution.

PA stenosis level in the TOF disease is widely varied from patient-to-patient; starting from mild pulmonary stenosis to pulmonary atresia with hypoplastic or absent pulmonary arteries.^17^,^18^ In mild cases of the PA stenosis, to avoid surgical intervention, one treatment strategy could be the use of cardiac catheterization with balloon angioplasty, occasionally with an arterial stent.^14^,^28^,^34^ Indeed, for mild stenosis levels, our simulations support the catheterization approach since post-operative stenosis recovery level is not high for the standard patch surgery. On the other hand, surgical intervention is preferred if the percentage of the PA stenosis is more than 50%, essentially the post-op improvement limit as shown in this paper. Most importantly, having defined the main performance parameters of patch repairs, our approach would aid the decision-making process for the borderline stenosis levels on a patient-specific basis.

The *Baseline* case employed in this study has been selected to represent the standard clinical operation. Briefly, a standard patch reconstruction starts with the measurement of main and branch PA sizes that are normalized by the body surface area (BSA) of the patient or descending aorta size at diaphragm level. Then, the length of the patch is determined by measuring the length of the incision from the right ventricle to the pulmonary artery, and its width is determined by visually holding the edges of the incision open at valve level and judging the size of the roof required to create a new pulmonary annulus with a diameter no larger than three-quarters (3/4) the diameter of the ascending aorta.^24^ The abnormal pulmonary vasculature is reconstructed in 3D using native tissue (treated or fresh pericardium) or artificial materials (PTFE/Dacron graft). Generally, a trans-annular patch should not be placed when the z-score value^7^ is larger than − 3. Otherwise, the incision is carried across the annulus, the pulmonary valve excised, and the patch inserted.^24^ Having a clinically realistic *Baseline* case is critical to evaluate and compare the performance of improved surgical alternatives.

In this paper, both the mechanical and hemodynamics performance parameters that can be considered to optimize patch design are established. These parameters are strongly influenced through the incision length and shape, the number of cuts, patch material, stenosis level and the post-operative lumen pressure. In comparing the different cut length models, the longer cut model (*Length_2*) exhibits the lowest post-operative stenosis of 13% compared to 24% and 32% in *Baseline* and *Length_1* cases respectively. Although case *Length_2* has slightly higher maximum stress on the patch (140 kPa), it has the minimum average stress level (35 kPa), which makes it more favorable. The shorter cut model (*Length_1*) experiences the lowest maximum stress on the model (81 kPa), but from a cross-sectional enlargement point of view, stenosis is not resolved fully, particularly at the patch corners (32% stenosis is remaining).

In relation to the cut shape, the oblique cut model (*Shape_1*) presents unfavorable differences compared to case *Baseline* and forms deformation hot spots (bumps) on the patch. These distortions disturb the blood flow and may also cause folding of the patch in the long term. Furthermore, average stress (72 kPa) and maximum stress (268 kPa) levels on the patch and the post-operative stenosis (34%) are substantially higher for the oblique cut model compared to the straight cut model. Thus, the present findings recommend that surgeons should make the incision as straight as possible, for relatively straight vessels.

For the proposed novel patch templates, having double-cuts (*Shape_2*), the average stress on the patch (216 kPa) is 160% higher than the corresponding value of the *Baseline* case (119 kPa). However, reconstruction of the artery through this method results very good lumen enlargement and post-op rotational symmetry. Still, the small size pediatric vasculature and the difficulty in reverse side access will limit future clinical adoption.

Patch material selection and its thickness influence the post-operative mechanics significantly. Among the simulated cases with different materials, the maximum stress on the patch varies significantly between 119 and 325 kPa. The PTFE material (*Baseline*) resulted the lowest peak stress (119 kPa) and the most homogeneous stress distribution around the stitch area compared to the other surgical material options. This is due to its high thickness and small Young’s modulus of elasticity. Whereas, the porcine xenopericardium, which is the thinnest and second stiffest material, resulted multiple local bumps (5.7 mm total deformation) and the highest stress concentration on the patch corners (325 kPa). Even though the human pericardium (*E* = 3.40 MPa) is stiffer than the porcine xenopericardium (*E* = 2.89 MPa), it has lower stress levels due to the fact it is 0.1 mm thicker than the porcine xenopericardium. Overall, using different surgical materials for the patch results in similar total deformation distributions and the highest deformation regions are localized proximal to the corner sides of the patch (Region 1 in Fig. 5(b)).

Pre-operative stenosis level also affects the post-operative patch shape, and stress level on the patch. Case *Stenosis* with 80% initial stenosis level is comparable to the *Baseline,* which has an initial stenosis level of 70%. To recover this higher stenosis level, the surgeon needs to open the slit as much as the *Baseline* case (16 mm gap width). This results in higher maximum stress (339 kPa compared to 881 kPa) on the artery intra-operatively, maybe due to higher curvature on the initial stenosis geometry. While the native tissue experiences this high stress level for a short duration, it could have a substantial effect for more extreme cases of stenosis. Further stretching of the tissue to open-up an appropriate gap for patch implantation would damage the native artery due to increased stress levels. Therefore, for extreme stenosis levels (higher than 80%), the replacement of the entire stenosis region with an artificial uniform diameter conduit can be justified for some patients.

Post-operative lumen pressure, which is typically between 25 to 45 mmHg, would influence the mechanical performance of pulmonary artery reconstruction. According to our simulations a 20 mmHg increase in post-operative pressure leads to 110% higher maximum stress levels on the patch and 7% more enlargement in vessel. These findings indicate that the post-operative pressure needs to be considered while designing the patch, especially in terms of maximum stress level rather than the stenosis recovery (Supplementary Fig. 1).

A number of important components of the present framework could be improved further. For example, determination of the initial patch shape is possible through multiple ways. For this research study, we implanted a cubic-wrapped patch with the same curvature as the surrounding native tissue and zero initial stress. This choice is reasonable since all initially planar linear elastic shells (raw patch materials) form a third-order cubic surface when subjected to bending load. Notwithstanding, the slight residual stresses are introduced when sheet patch materials are bended from flat sheets.

Likewise, simulations with initially flat and extremely cambered patches are also performed (Supplementary Fig. 2). These simulations showed that increasing the surface curvature of the initial patch do not affect the peak stress value but it alters the location of high stress regions from the patch corners towards the lateral sides. Actually, the initial graft configuration is highly dependent on the initial slit opening area. Thus, generating equally curved patches for different surgical scenarios may not be the optimal strategy. For example, in double patch case, the initial configuration of the opened gaps constrains the use of a relatively flat “tangent” patch, since implanting two bulgy patches instead would lead to the vessel dilation for the post-operative state. Likewise, for the shorter cut case using a flat patch would not resolve the stenosis problem effectively, and a “tangent” patch (which is relatively bulgy) is preferred for this case. Therefore, in our framework by keeping the patch generating method fixed instead of the curvature level, unreasonable patch options are eliminated. This approach also allows a more unbiased comparison. It is worthwhile to mention that bulgier patches may be associated with flow recirculation and potentially flow stagnation zones.

Another approach for determining the initial patch shape is through rapid prototyping (RP); following the registration/scaling step, the initial patch shape predicted by the FEM can be printed and the desired patch shape can be extracted directly from the printed model using a healthy RP model as a master with the help of the surgeon. This approach is partially illustrated in our experimental validation study (Supplementary Material1). Finally, the complex 3D patch shapes predicted by the present approach can be developed through a 2D flattenization code and these shapes can be cut from the flat raw patch materials prior to implantation.

Another limitation of the current study, is the number of parameters being varied concurrently, which somewhat challenges the comparison across cases. This includes the geometrical variations in patch parameters (beyond incision shape), which challenges performance comparison across geometrical variations (Length and Shape), but also concurrent variations in mechanical properties and patch thickness. Further modeling limitations may include the use of a Newtonian blood flow model. This approach is justified in the pulmonary artery due to its large size. Furthermore, all the cases are compared within each other, thus the non-Newtonian effect is expected have relatively equal influence. Although structural and hemodynamics analysis have been performed in the current study, modeling the fluid structure interaction (FSI) has been left as a future work. This is also justified from comparative perspective and it is expected to bring roughly 10% difference in hemodynamic results.

Surgical patch reconstruction of pulmonary artery stenosis is a complex procedure. This task is carried out skillfully during cardiopulmonary bypass, without blood flow inside the vessel lumen. The unpressurized stage of surgery makes it particularly challenging to predict the best post-operative patch size and shape to reconstruct the stenosed vessel zone. While there are conventional rules of cardiovascular surgeries, computer simulations may also enable us to test novel designs and provide a mechanism for creativity through virtual surgeries without any harm to the patient. In this study, we present a framework to demonstrate the post-operative performance for a number of surgery parameters; cut configuration of the artery, patch material and hemodynamic indices. Based on these results, a longer cut with PTFE patch material (*Length_2)* appears be the preferred case for real surgeries compared to other cases presented in this study due to its better stenosis recovery function, especially for vessels with very high stenosis level. Straight cut is preferred to oblique cuts, which cause relatively larger bulbous regions and result in lower stenosis recovery. Another critical finding relates to the thickness of the patch and how changing the thickness of the patch significantly affects the post-operative performance of the patch more than the material parameters. The patch material property is of secondary importance and it seems more suitable to use a patch with a lower stiffness, but not lower than the vessel tissue. Although it might cause higher maximum stress, our novel double patch configuration appears to useful for cases of very high stenosis, where a single cut and single patch might not be enough to recover the stenosis level.

Based on our preliminary experience with ongoing patient specific geometries the patch surgical planning would take approximately 1 week for the generation of surgical recommendations from the time of receiving 3D reconstruction of the pre-surgical MRI data. By applying the preoperative computed planning to more complex congenital cardiac reconstructive surgeries, we may reduce the early and late postoperative complication rate and thus improve mortality of the children who undergo cardiac surgery.

## Electronic supplementary material

Below is the link to the electronic supplementary material.

Supplementary material 1 (DOCX 1687 kb)

Supplementary material 2 (DOCX 1457 kb)

Supplementary material 3 (PPTM 1970 kb)
