# Supplementary material for: Computational Pre-surgical Planning of Arterial Patch Reconstruction: Parametric Limits and In Vitro Validation
Source: Ann Biomed Eng. 2018 May 14;46(9):1292–308. doi: 10.1007/s10439-018-2043-5 (PMC6097742; doi:10.1007/s10439-018-2043-5)
Supplement: Supplementary file 1 — Supplementary material 1 (DOCX 1687 kb) [file 10439_2018_2043_MOESM1_ESM.docx]

**Computational pre-surgical planning of arterial patch reconstruction – parametric limits and in vitro validation**

S. Samaneh Lashkarinia^1^, Senol Piskin^1, 2^, Tijen A. Bozkaya^3^,

Ece Salihoglu^4^, Can Yerebakan^5^, Kerem Pekkan^1^

^1^ Department of Mechanical Engineering, Koc University, Istanbul, Turkey.

^2^ Department of Mechanical Engineering, University of Texas at San Antonio, Texas, USA.

^3^ Department of Cardiovascular Surgery, Koc University Medical School, Istanbul, Turkey.

^4^ Department of Cardiovascular Surgery, Istanbul Medipol University, Istanbul, Turkey.

^5^ Cardiovascular Surgery, Children’s National Heart Institute, The George Washington University School of Medicine, Washington DC, USA

**Supplementary Computational Model Validation**

**1. Introduction**

V*erification* of the finite element solver used in the proposed patch-planning framework is detailed in the main manuscript text (Please see Methodology and Discussion Sections). These verification studies include mesh convergence analysis, testing different boundary condition schemes, thickness and material property sensitivity analysis. However, full model *credibility*, requires an experimental *validation* campaign, so that clinicians and scientists can confidently extrapolate the information and decisions based on the present model predictions [^1^](#_ENREF_1). Therefore, in this section, a novel finite element model validation approach that is based on rapid-prototyping is presented.

**2. Experimental set-up**

Initial geometry of the stenosed vessel is built by rapid prototyping (Form2, Formlabs Inc, MA, USA). 3D printed experimental main pulmonary artery (MPA) replica is manufactured from a flexible resin material (Formlabs Inc, MA, USA) having 50 µm layer thickness precision. The entire printing process takes around 9 hours. Cleaning step is performed in alcohol for 2 minutes (99% Isopropyl) in order to replicate vessel material properties. The test case is then cured under 80 mW/cm2 of 365 nm fluorescent light for 15 minutes -see Figure 1 (a).

The sequence of intra-operative surgical steps is performed on the rapid-prototype diseased MPA replica exactly as in the real surgery. First, the surgical incision is introduced on the rapid-prototype MPA by the pediatric cardiovascular surgeon (Dr. Ece Salihoglu) - see Figure 1 (b). Geometrical parameters of the incision are the same as the *Baseline* computational model case (straight cut – 50 mm length). A 1 mm thickness PTFE tissue (GORE-TEX Soft Tissue Patch, W. L. Gore & Associates Inc, AZ, USA) is then used for the patching operation. In order to replicate the 3D patch shape exactly as in to the computer-generated model, this patch geometry is also 3D-printed from the flexible resin material and used by the surgeon as a pattern during the cutting and shaping operation of the experimental PTFE patch - see Figure 1 (c) and (d). The vessel test case is then opened (same gap as in the computational model) and the patch is fitted in to the stretched slit opening area. Body and patch are then completely bonded using Super Glue (cyanoacrylate) - see Figure 1 (e).

For the static pressure test, a stopper is attached to the 3D printed and patched prototype outlet and the nitrogen gas is supplied in to the vessel from the other end. A similar set-up is utilized in a recent study that is conducted by our group [^2^](#_ENREF_2). Experimental measurements are acquired when the desired intramural pressure level is reached (30 to 90 mmHg pressure range). Lateral deformations of the model are recorded using a high-speed camera in 2D. The diameter of the vessel is measured using a motion analysis software (Maxtraq, Innovision Systems Inc, MI, USA).

The flexible resin material properties are measured *in house* due the strong effect of the post-curing duration. Uniaxial mechanical tests are conducted both along the tangent and perpendicular directions of the structural layout layers of the 3D printed flexible resin sheet having the same thickness as the MPA model. Stress-strain curves are obtained by sinusoidal stretching of rectangular shaped samples (10 x100 mm) up to 20% in axial directions, using two linear motor configurations in the BOSE planar test system (BOSE, Framingham, Massachusetts). Since the material properties of the resin depends significantly on the post-curing time and the time passed after printing, the experimental test sample is built and tested in the same condition and age as the test case.

The use of super-glue on the flexible resin and the PTFE tissue alters the material properties of both materials. Thus, to obtain the correct material properties at the bonded regions, thin layer of the glue is placed on the resin (10 x 100 mm) and PTFE (10 x 10 mm) samples and mechanical tests are performed. The protocol to obtain the material properties from biaxial tests are provided in Section 2.4 and employed here as well. The linear elastic material properties of the materials utilized in this experimental campaign are presented in Table 1.

Finite element model of *Baseline* case as defined in Section 2.3 of the manuscript is employed to obtain computational deformations corresponding to the experimental pressure levels. As in the experimental set-up, both end section boundary conditions are specified as fixed in all directions. Intramural pressure loading is increased from 0 to 90 mmHg. Material properties of the experimental model are assigned from the mechanical test measurements.

**3. Results**

The diameter of the patched vessel at the final loading configuration (90 mmHg) is provided in Figure 2 for both experiments and computations. The change in deformation between the initial (0 mmHg) and final configuration (90 mmHg) is plotted in Figure 2 (a). Experiments are repeated 5 times for 3 pressure load levels, and the corresponding diameters are recorded. An average diameter change of 0.82 mm, 1.8 mm and 3.12 mm corresponding to 30 mmHg, 60 mmHg and 90 mmHg pressure conditions are measured respectively. The computed deformation contour projected on the lateral side of the vessel is displayed in Figure 2 (b). All loading states are presented in Figure 3 where the experimental and computational average diameter values are compared. Percent error is defined as the ratio of the difference between the experimental diameter and its corresponding computational model diameter relative to the experimental diameter value. According to this error description, error range is 1-3 % in all simulations.

**4. Discussion**

An alternative to the present *in vitro* experimental approach is to use *ex vivo* vessel segments from animals in the validation tests. In this approach it is almost impossible to obtain a diseased vessel having a stenosis. Therefore, we utilized flexible rapid prototyping to build a vessel with stenosis. While the alternative options of the appropriate flexible materials for the present validation campaign are found to be limited, the flexible resin (Formlabs Inc, MA, USA) served our purpose relatively well. One particular challenge related to the flexible resin material is its low tear resistance. This property resulted poor surgical suture retention during the stitching and caused leaks around the implanted patch. Silicone elastomer materials can be a better substitute to flexible resin and will be employed in future studies.

Due to the symmetry of the MPA model only the deformations from the lateral side are compared. Future studies on complex patch reconstructions in patient specific anatomies will consider capturing the 3D strain distributions using two cameras following the present computational analysis on idealized geometry. Performing fluid-structure interaction analysis will also be worth investigating in patient specific patch cases. For a comprehensive validation of the present surgery-planning framework, we have proposed an animal study to be carried out on the carotid arteries of rabbits with the use of advanced microsurgical techniques. For this study, almost 60 days old rabbits undergo patch implantation surgeries and will be monitored for surgery performance and growth of the vessel. This experimental study will help us to verify and validate the current computational study in more details.

**5. Acknowledgements**

We thank Erhan Ermek for major assistance with pressure and material mechanical tests.

**6. References**

1. Henninger H. B., S. P. Reese, A. E. Anderson and J. A. Weiss. Validation of Computational Models in Biomechanics. *Proceedings of the Institution of Mechanical Engineers. Part H, Journal of engineering in medicine* 224: 801-812, 2010.

2. Oguz G. N., S. Piskin, E. Ermek, S. Donmazov, N. Altekin, A. Arnaz and K. Pekkan. Increased Energy Loss Due to Twist and Offset Buckling of the Total Cavopulmonary Connection. *Journal of Medical Devices* 11: 021012-021012-021018, 2017.

| **Test** | **Material** | | **young's modulus**  **(MPa)** | | **Poisson's ratio**  **(-)** | **Thickness**  **(mm)** |
| --- | --- | --- | --- | --- | --- | --- |
| ***Biaxial*** | ***PTFE - Glue*** | | 8.9 | | 0.45 | 1.00 |
| ***Uniaxial*** | ***Flexible resin*** | ***Tangential direction*** | 9.97 | 9.71 | 0.45 | 0.7 |
|  |  | ***Transverse direction*** | 9.45 |  |  | 0.7 |
|  | ***Flexible resin – Super glue*** | ***Tangential direction*** | 19.96 | 19.51 | 0.45 | 0.9 |
|  |  | ***Transverse direction*** | 19.07 |  |  | 0.9 |

**Table 1:** Linear elastic material properties and thickness of tested materials which are found in the validation test case.


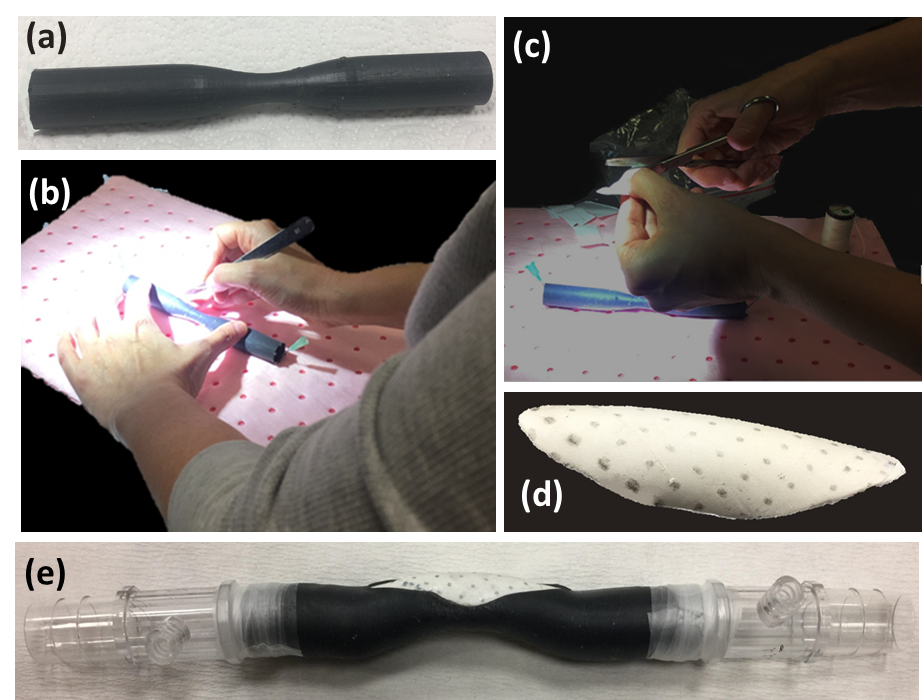


**Figure 1:** (a) Shows the initial rapid-prototype of 70% stenosed main pulmonary artery model manufactured from of flexible resin material. (b) The creation of the initial suture line on the test case by a pediatric cardiovascular surgeon. (c) Intraoperative shaping of PTFE patch with respect to existing computer-generated template of patch (*Baseline* case). (d) The implanted PTFE patch before the post-operative pressure loading experiments. (e) The final assembled patch model. Connections are attached to both ends to fix the model in experimental set-up and supply pressurized gas.


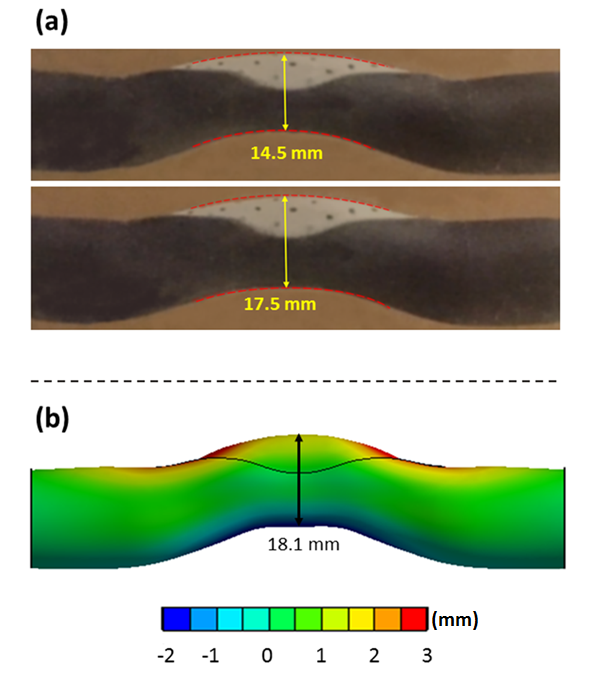


**Figure2:** (a) Upper image shows the test case in zero-pressure condition. Lower image shows the test case, which is pressurized to 90 mm-Hg loading by exerting nitrogen gas in to the vessel. (b) Distribution of the vertical deformation (y-axis) obtained from the finite-element model simulation. Boundary, initial conditions and material properties are as same as the experimental test case.


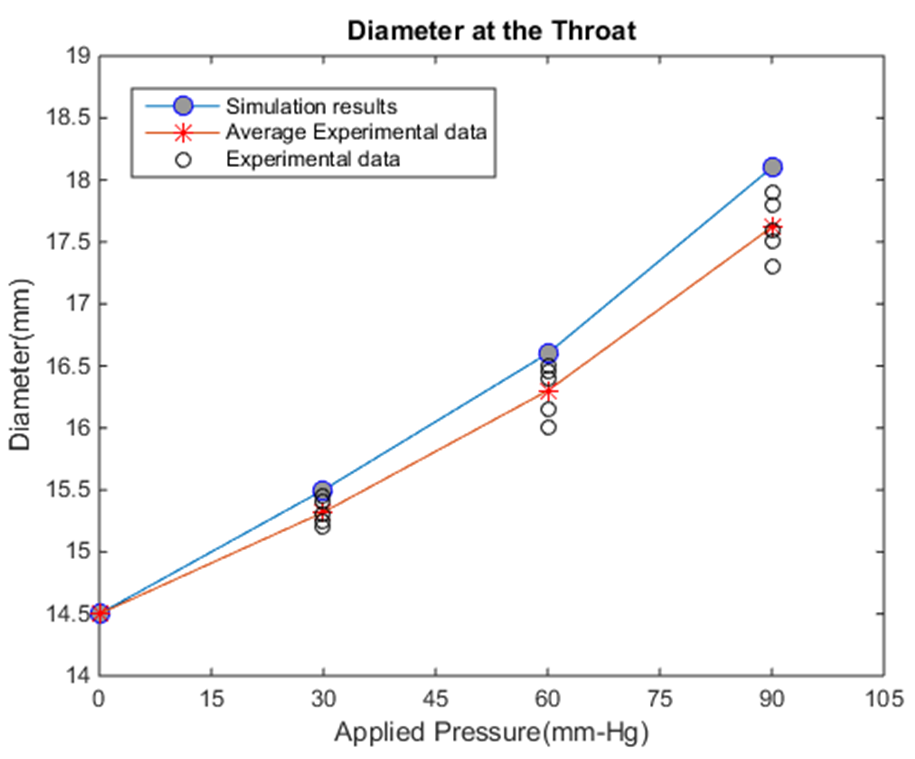


**Figure 3:** Comparison of the measured throat diameter with the computational model results for three different pressure loading conditions.
