# Supplementary material for: Computational Pre-surgical Planning of Arterial Patch Reconstruction: Parametric Limits and In Vitro Validation
Source: Ann Biomed Eng. 2018 May 14;46(9):1292–308. doi: 10.1007/s10439-018-2043-5 (PMC6097742; doi:10.1007/s10439-018-2043-5)
Supplement: Supplementary file 2 — Supplementary material 2 (DOCX 1457 kb) [file 10439_2018_2043_MOESM2_ESM.docx]

**Computational pre-surgical planning of arterial patch reconstruction – parametric limits and in vitro validation**

S. Samaneh Lashkarinia^1^, Senol Piskin^1, 2^, Tijen A. Bozkaya^3^,

Ece Salihoglu^4^, Can Yerebakan^5^, Kerem Pekkan^1^

^1^ Department of Mechanical Engineering, Koc University, Istanbul, Turkey.

^2^ Department of Mechanical Engineering, University of Texas at San Antonio, Texas, USA.

^3^ Department of Cardiovascular Surgery, Koc University Medical School, Istanbul, Turkey.

^4^ Department of Cardiovascular Surgery, Istanbul Medipol University, Istanbul, Turkey.

^5^ Cardiovascular Surgery, Children’s National Heart Institute, The George Washington University

**Supplementary Material: Mechanical Characterization of Materials Used in Pediatric Surgical Reconstructions**

## 1. Sample preparation and mechanical tests

Sections of the material specimens were cut out from the actual 20 mm diameter PTFE and Dacron conduits that are commercially available for pediatric cardiovascular surgeries. The porcine pericardium (Edward Lifescineces, Irvine, CA) in 20x90 mm size was kept in glutaraldehyde solution as in the standard surgical protocol and immediately tested. Likewise, a discarded sample of human pericardium (20x50mm) originally prepared for surgical implantation is tested with in the same day through approved IRB protocol.

Biaxial mechanical tests are conducted for each material using four linear motor configurations in the BOSE planar biaxial test system (BOSE, Framingham, Massachusetts) by sinusoidal stretching of square shaped samples (10x10mm) up to 20% in both axial directions (Supplementary Figure 1). Preconditioning cycle at 0.1 Hz started after removing the slack and zeroing the cell value in both axis then load cells were zeroed in the software. Preconditioning step covered the first 50 load cycles of the experimental protocol and this data set was not used in analysis. Each experimental load cycle includes 50 data points (25 for loading and 25 for unloading per sample) and a total 100 cycles were conducted for each material sample. Strains are measured in both directions through a camera tracking the tissue dye-marked fiducial points as well as linear motor positions.

**2. Poisson’s ratio and Young’s modulus**

Average Young’s modulus values corresponding to the load ranges encountered in patch reconstructions are computed from the planar biaxial tensile test data. For this purpose, the two-dimensional stress formulas that govern the planar biaxial tensile test configuration are used;

$\sigma_{x}=\frac{E\left(\varepsilon_{x}+\nu\varepsilon_{y}\right)}{1-\nu^{2}},\sigma_{y}=\frac{E\left(\varepsilon_{y}+\nu\varepsilon_{x}\right)}{1-\nu^{2}}$ (1)

Where *σ_x_, σ_y_* and *ε_x_, ε_y_* are the engineering stress and strain components in Cartesian coordinates, respectively. These stress and strain components are the arithmetic means of the measured force-displacement data via the following relations;

$\sigma_{x}=\frac{F_{x}}{tl_{y}},\sigma_{y}=\frac{F_{y}}{tl_{x}}$ (2)

Where *t* is the sample thickness that is obtained through an optical coherence tomography system (Thorlabs Inc, NJ, USA) at 0.5 μm resolution as discussed in the manuscript text. *l_x_* and *l_y_* are sample dimensions which both are ~10mm in all tests.

The Young’s modulus is computed through Eq. (3) based on a linear regression fit to the sums of the average stress and strain values;

$\sigma=\sigma_{x}^{'}+\sigma_{y}^{'}=\frac{E\left(w_{1}\varepsilon_{x}+w_{2}\varepsilon_{y}\right)}{1-\nu}=\frac{E\varepsilon}{1-\nu}$ (3)

Where $w_{1}$ and $w_{2}$are weighting factors.

Finally the Poisson’s ratio values are estimated from the following approximation via linear regression.

$\nu\approx\frac{\Delta L_{y}}{\Delta L_{x}}$ (4)

**3. Stress-Strain data and computed linear elastic model material properties**

Linear regression is implemented in biaxial tensile test measurements as described in Equation (3) and (4), in order to compute Poisson’s ratio and Young’s modulus, respectively. In Figure 2, stress-strain data for all materials in both orthogonal directions are presented. Weighting factors for the stress function, Equation (3) are selected and kept the same for each material to minimize the residual error in the objective function. All specimens are stretched up to ~%10 of their original length in order to investigate their linear response of hyperelastic materials. Computed Poisson’s ratio and Young’s modulus values in whole range for PTFE, Dacron and porcine pericardium are given in Table 2 of main text. The reported mechanical properties of materials used aortic arch reconstruction, by Tremblay et al. agree well with the reported stiffness range^2^.

**4. Discussion**

Although the material model used in this study represents the physics faithfully well in the operating loading range, there are several limitations for further development. First of all, instead of using linear elastic material properties in the simulations, strain energy density function material models can be used for the patch and artery to better describe their nonlinear and time-dependent material properties. While more complex material models can easily be implemented, and the corresponding biaxial material property data is available in-house, for this manuscript the material model is kept simple so that it can provide us the physical insight on the relative material properties of patch and native tissue, providing comparative information on different patch strategies.

Biaxial tensile testing is an established technique to determine the mechanical properties of biomedical materials, which can provide both the linear and nonlinear elastic characteristics. Obviously, hyperplastic materials demonstrate linear response at small stresses and become nonlinear as the applied force increases. Therefore, average stiffness of hyperplastic materials can be computed by using linear model at small stresses. Besides, homogeneity and isotropy are other approximations to the realistic material in order simplify the case and provide rough estimations about the stiffness of the material where, in fact, the material itself may be nonhomogeneous and anisotropic up to an acceptable degree. A detailed nonlinear mechanical characteristics of these materials will be presented in a future publication^1^.

**5. References**

1. Donmazov S., Piskin, S., Ermek, E., and Pekkan, K. Mechanical Characterization and Torsional Buckling Effects of Vascular Conduits,. *J. Mech. Behav. Biomed. MaterJ. (to be submitted)* 2016.

2. Tremblay D., T. Zigras, R. Cartier, L. Leduc, J. Butany, R. Mongrain and R. L. Leask. A Comparison of Mechanical Properties of Materials Used in Aortic Arch Reconstruction. *The Annals of Thoracic Surgery* 88: 1484-1491.


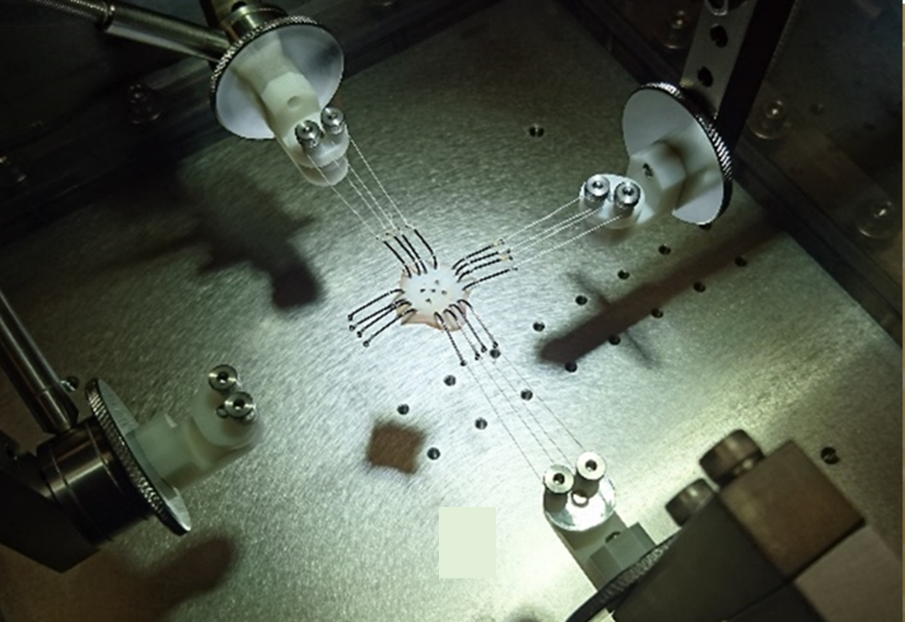


**Supplementary Figure** 1: Biaxial tensile test setup with the PTFE specimen oriented through fish-hook support system**.**


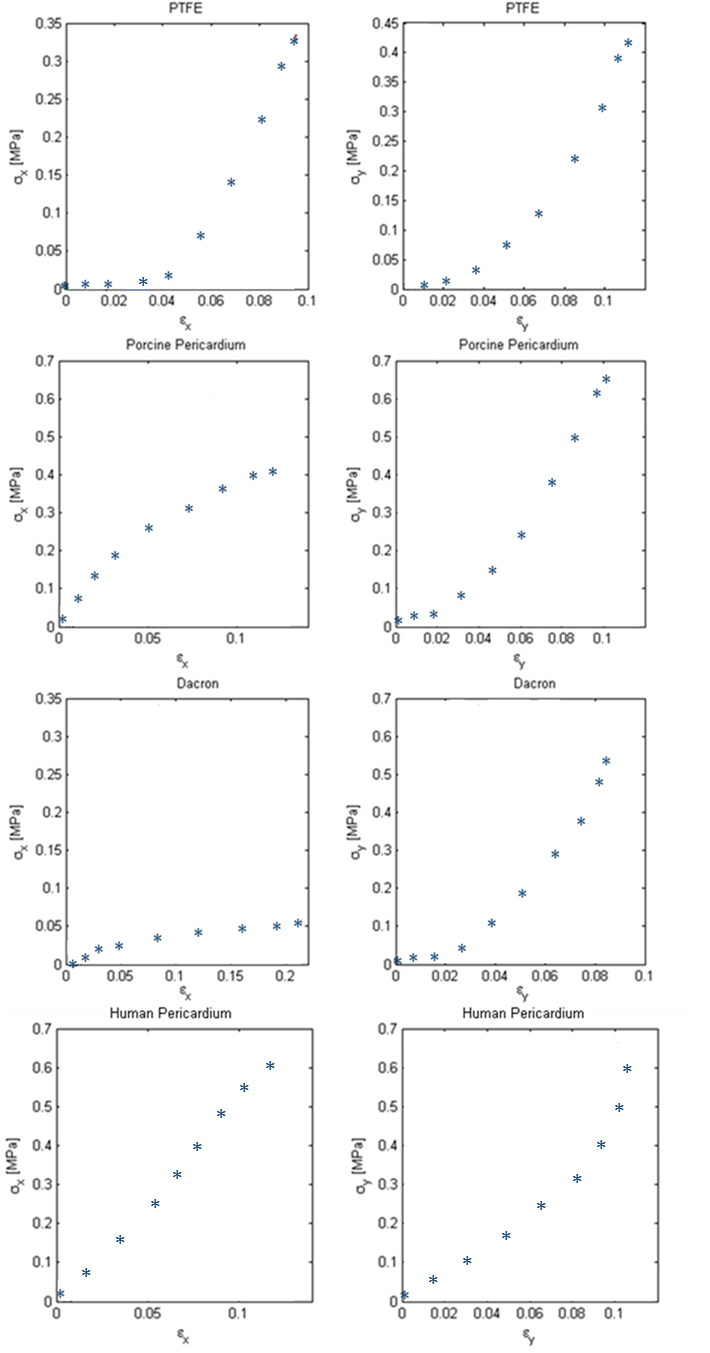


Supplementary Figure 2: Experimental stress-strain data for pediatric surgical materials; PTFE, Dacron, porcine and human pericardium represented by stars obtained from force-net displacement biaxial tensile test measurements.
