# Supplementary material for: Computational Pre-surgical Planning of Arterial Patch Reconstruction: Parametric Limits and In Vitro Validation
Source: Ann Biomed Eng. 2018 May 14;46(9):1292–308. doi: 10.1007/s10439-018-2043-5 (PMC6097742; doi:10.1007/s10439-018-2043-5)
Supplement: Supplementary file 3 — Supplementary material 3 (PPTM 1970 kb) [file 10439_2018_2043_MOESM3_ESM.pptm]

## Slide 1
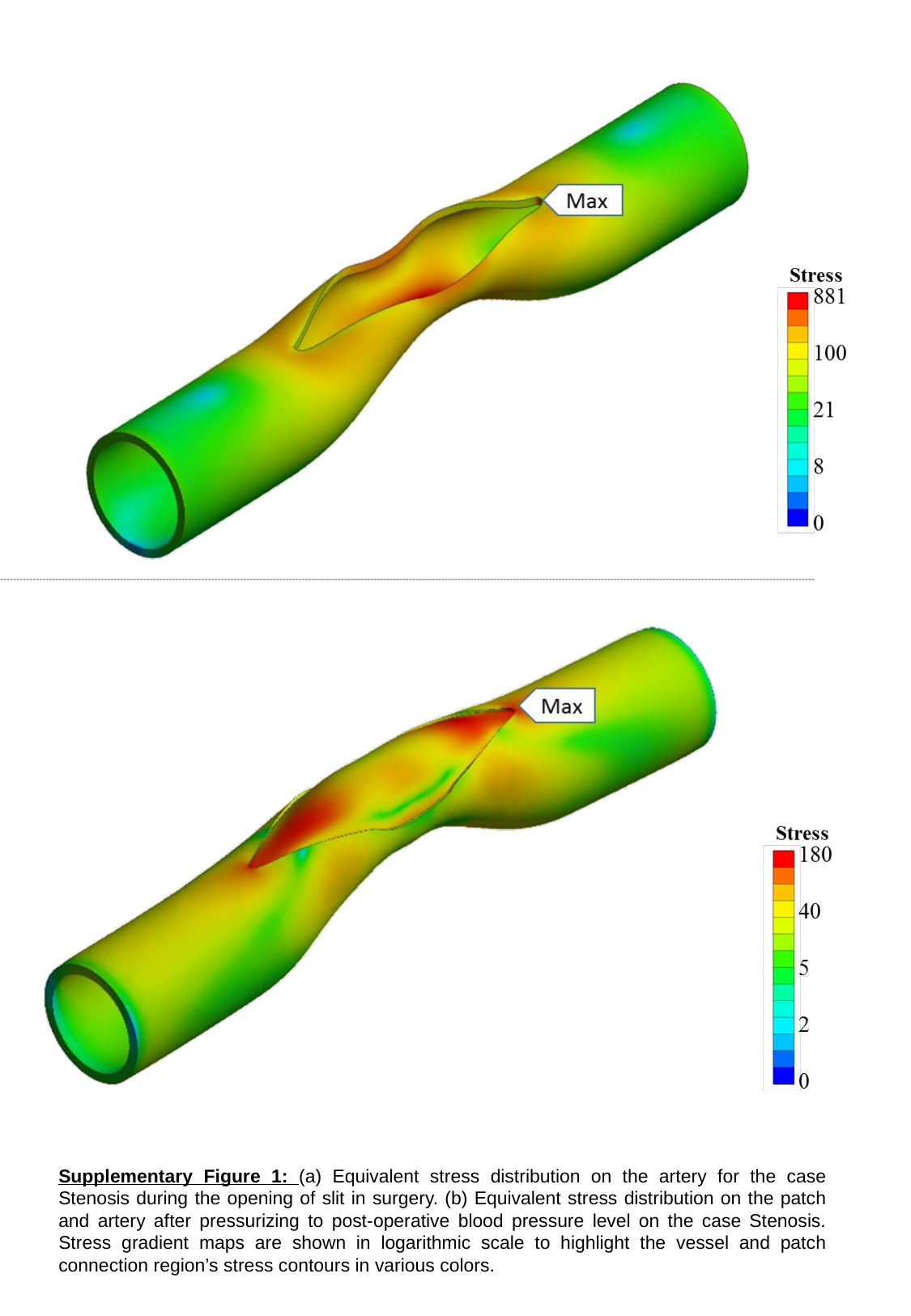

Supplementary Figure 1: (a) Equivalent stress distribution on the artery for the case Stenosis during the opening of slit in surgery. (b) Equivalent stress distribution on the patch and artery after pressurizing to post-operative blood pressure level on the case Stenosis. Stress gradient maps are shown in logarithmic scale to highlight the vessel and patch connection region’s stress contours in various colors.

## Slide 2
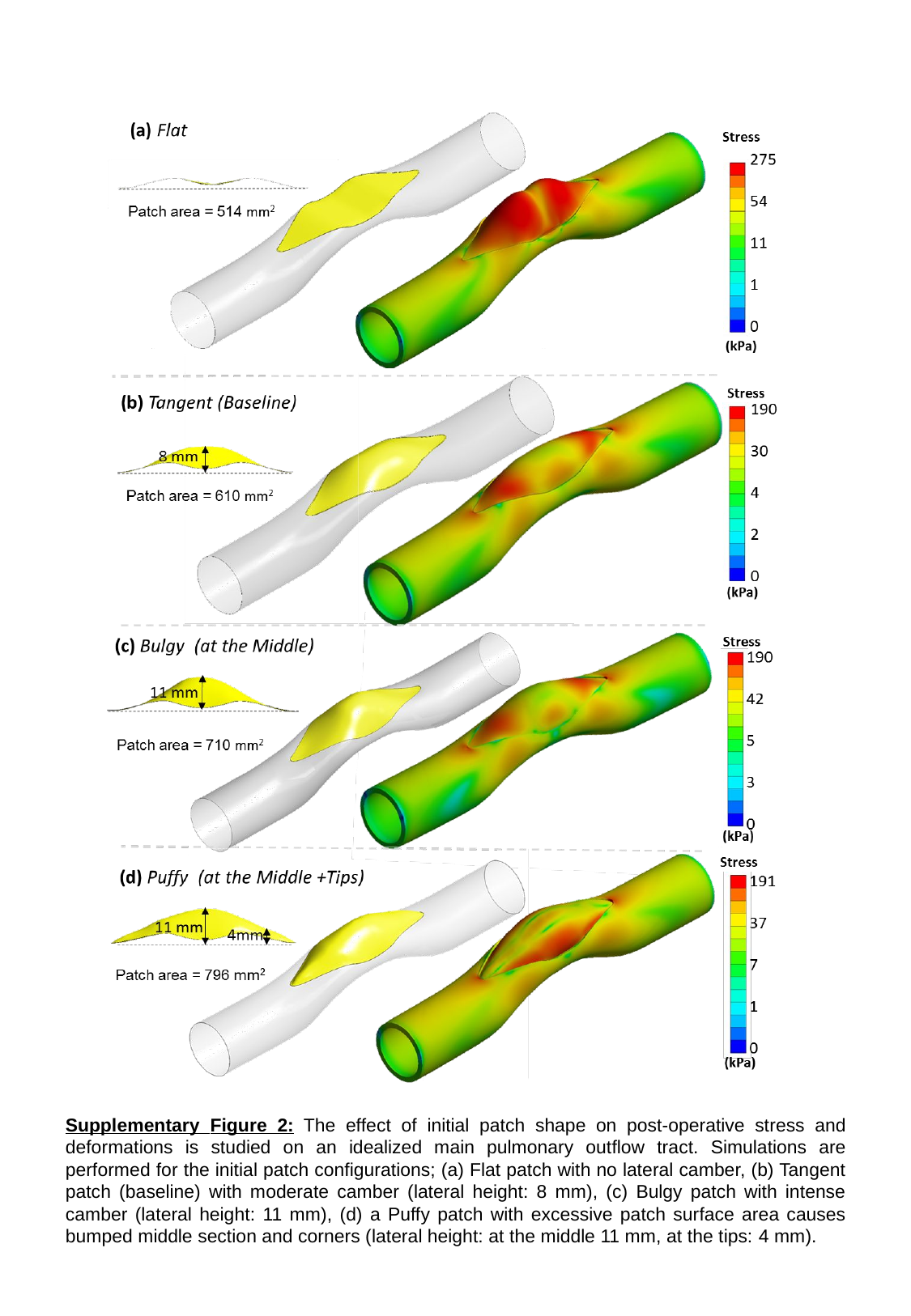

Supplementary Figure 2: The effect of initial patch shape on post-operative stress and deformations is studied on an idealized main pulmonary outflow tract. Simulations are performed for the initial patch configurations; (a) Flat patch with no lateral camber, (b) Tangent patch (baseline) with moderate camber (lateral height: 8 mm), (c) Bulgy patch with intense camber (lateral height: 11 mm), (d) a Puffy patch with excessive patch surface area causes bumped middle section and corners (lateral height: at the middle 11 mm, at the tips: 4 mm).
